# Inflammation in Schizophrenia: The Role of Disordered Oscillatory Mechanisms

**DOI:** 10.3390/cells14090650

**Published:** 2025-04-29

**Authors:** Lucinda J. Speers, David K. Bilkey

**Affiliations:** 1 Grenoble Institut of Neurosciences–Inserm, 3800 Grenoble, France; lussejean@gmail.com; 2 Psychology Department, University of Otago, Dunedin 9016, New Zealand

**Keywords:** inflammation, IL-6, oscillations, GABA, schizophrenia, hippocampus, synchrony, theta, gamma, phase precession

## Abstract

Schizophrenia is a chronic, debilitating disorder with diverse symptomatology, including disorganised cognition and behaviour. Despite considerable research effort, we have only a limited understanding of the underlying brain dysfunction. A significant proportion of individuals with schizophrenia exhibit high levels of inflammation, and inflammation associated with maternal immune system activation is a risk factor for the disorder. In this review, we outline the potential role of inflammation in the disorder, with a particular focus on how cytokine release might affect the development and function of GABAergic interneurons. One consequence of this change in inhibitory control is a disruption in oscillatory processes in the brain. These changes disrupt the spatial and temporal synchrony of neural activity in the brain, which, by disturbing representations of time and space, may underlie some of the disorganisation symptoms observed in the disorder.

## 1. Introduction

When immune system activation, and the resulting inflammatory response, occurs during critical periods of brain development, it can significantly influence the risk and progression of several conditions, such as autism spectrum disorder (ASD), attention-deficit/hyperactivity disorder (ADHD), bipolar disorder, microcephaly, cerebral palsy, and schizophrenia [1]. While a considerable body of work describes how inflammation can alter brain connectivity, particularly in early development, it is less clear how these changes result in some of the complex symptomatology observed in diseases such as schizophrenia. This review will describe data that provide one possible link, with a particular interest in how inflammation-mediated changes in GABAergic mechanisms could disrupt the synchrony and coherence of spatial and temporal processing in the brain. Although the focus is on schizophrenia, the underlying mechanisms will likely overlap with several other neurodevelopmental conditions.

## 2. Evidence of Altered Inflammatory Responses in Individuals with Schizophrenia

Schizophrenia is a chronic, severe, disabling brain disease that is among the world’s top ten causes of long-term disability [2], affecting approximately 1% of the population across a lifetime. The symptoms of schizophrenia are typically separated into three broad categories: psychotic or positive symptoms, negative symptoms, and cognitive impairment [3]. The overt positive symptoms consist of hallucinations and delusions; the negative symptoms include problems with motivation and social interactions; while the cognitive symptoms comprise deficits in attention, memory, and executive function. The cognitive symptoms are recognised as a fundamental feature of the disorder, and their increased intensity is strongly related to negative social and vocational outcomes [4,5,6]. Current evidence indicates that schizophrenia results from a complex interplay of genetic, biological, and environmental factors, including epigenetic mechanisms that converge on shared pathways of molecular dysfunction. The primary risk factors appear to be subtle and accumulative, suggesting that initial events may trigger a developmental cascade of secondary events that progressively enhance schizophrenia risk. One risk factor that has received considerable attention in recent years is inflammation, and in particular, the effects of the cytokines that are secreted by the immune system as part of the inflammatory response to pathogens or injury, including to non-physical insults such as stress and depression [7,8].

Elevated cytokine levels have frequently been observed in the blood and cerebral spinal fluid of schizophrenia patients, including alterations in the inflammatory cytokine interleukin-6 (IL-6), Interleukin-1 beta (IL-1β), Tumour Necrosis Factor alpha (TNF-α), Interferon-gamma (IFN-γ), and the chemokine Monocyte Chemoattractant Protein-1 (MCP-1). This suggests that inflammatory processes may have a causal role in the disease or that they are a downstream consequence of the disorder. The fact that abnormalities in cytokine levels are also present in individuals experiencing their first psychosis episode and in the first-degree relatives of patients diagnosed with schizophrenia suggests that they may have a causal role [9,10]. Currently, some of the most robust cytokine findings are for IL-6, which has been described as a potential trait marker for schizophrenia in recent meta-analyses [11]. Higher IL-6 levels have been associated with higher scores on a childhood trauma questionnaire and with lower cognitive and social cognitive function [12]. Increases in IL-6 have also been associated with decreased executive function and poorer verbal learning and memory and attention processing [13]. In a recent meta-analysis, increased levels of a number of cytokines, including IL-6, were reported in individuals with either acute or chronic schizophrenia-spectrum disorder [14]. These data supported the results of earlier studies, which had described similar effects in neuroleptic naïve, first-episode psychosis patients [15]. Elevated cytokines are likely to underlie a number of downstream effects; for example, several studies have shown that IL-6 stimulates dopamine release, a key factor underlying positive symptoms, while dopamine itself exerts a reciprocal regulatory effect on inflammatory processes (for a review, see [16]). IL-6 levels have also been positively correlated with the negative and cognitive symptoms of schizophrenia, potentially via its effects on NMDA receptors and GABAergic interneurons [17].

## 3. The Role of Cytokines in the Aetiology and Development of Schizophrenia

Elevated cytokine levels during critical developmental periods, including in the prenatal environment, and during adolescence have been associated with the disorder and may contribute to the neurodevelopmental component of the disease [18]. Support for this hypothesis initially came from studies showing that an association exists between schizophrenia risk and increased exposure to pathogens or maternal adversity, such as being born during winter or spring, living in urban environments (particularly inner city neighbourhoods of low socio-economic status) or migrant communities, exposure to childhood trauma and social distress, and drug abuse, all factors that are associated with infection and/or inflammation [19,20]. Although many of these environmental risk factors can exert effects throughout childhood and early adulthood, most studies have identified two critical periods of development that are particularly sensitive to environmental insults—first, the pre- and perinatal period, and second, the adolescent period [18,21,22]. The idea that impacts during both of these periods might be important has been conceptualised as the two-hit hypothesis, whereby an initial event during early neurodevelopment (the first hit) induces subtle impairments that may not reach clinical significance by themselves but which instead predispose the individual to increased vulnerability to stressors occurring later in life (the second hit) [23]. In support of this proposal, evidence of broad cognitive and structural abnormalities among high-risk youth before the onset of psychosis suggests that a predisposing event has occurred early in development [24,25,26]. Furthermore, this hypothesis is supported by extensive epidemiological studies of schizophrenia patients and their families, which show that prenatal environmental factors such as famine or infection, particularly occurring during the first or second trimesters, are involved in the aetiology of the disorder [27,28,29,30,31,32,33]. For the infection risk factor, it has been demonstrated that it is the release of proinflammatory cytokines during the maternal immune response to pathogens, rather than prenatal exposure to the pathogens themselves, that triggers abnormal neurodevelopment in the foetus [34,35]. Maternal immune activation (MIA) studies show that inflammatory cytokines such as IL-6 can cross the placental barrier at critical moments of neurodevelopment [8,35,36,37]. Furthermore, maternal inflammation itself may indirectly trigger elevated levels of cytokines such as IL-6 in the placenta, without necessarily requiring transport across the placenta [38]. A number of recent papers review these MIA effects and how they are linked to schizophrenia and other disorders, e.g., [39,40,41,42].

Elevated cytokine levels in utero, occurring during key moments of gestation, can affect several developmental processes, including the proliferation, differentiation, and migration of neuronal subtypes to their appropriate locations, as well as the formation and connectivity of synapses [8,32,35]. This can lead to critical structural and functional abnormalities in the brain. For example, data from several different animal models have shown that MIA results in disrupted cytoarchitecture and molecular signalling pathways in MIA offspring, as well as behavioural abnormalities that match the symptomatic profile of schizophrenia [18,30,42]. In particular, elevated levels of cytokines induced by immune system activation during gestation have been shown to result in altered cortical layering [43] and neuronal signalling [44], which may enhance the effects of “second hits” [45,46] during later stages of development, such as adolescence.

Adolescence is generally defined as the time between the onset of puberty through to the mid-twenties, and is the period when widespread pruning of excess synapses occurs in the brain, with a subsequent myelination of surviving axons [47,48,49]. These processes underlie the maturation of the prefrontal cortex [50], a process that can continue into early adulthood [51]. Adolescence is, therefore, a critical developmental period when the ‘second hit’ may be most disruptive. Consistent with this, late adolescence is when the initial symptoms of psychosis are most likely to emerge, and numerous studies show reductions in cortical grey matter among high-risk adolescents who will then go on to develop symptoms of psychosis [52,53,54]. Recent studies have demonstrated how inflammation is associated with changes in several of the processes that are remodelling the brain during adolescence, for example, axon myelination [55] and synaptic pruning [56,57]. Cytokines such as TNF-α have also been shown to affect dopamine signalling by altering the function of dopamine receptors and the synthesis of dopamine itself. Since dopamine dysregulation is a hallmark of schizophrenia, this links inflammation to one of the prominent theories of the disease [58]. Cytokines have also been shown to interact with the glutamate system, which is involved in rapid communication in the brain, as well as in synaptic plasticity and cognition. For example, IL-1β and TNF-α can affect the function of the NMDA subtype of glutamate receptor, which is crucial for learning and memory. This interaction could contribute to cognitive deficits in schizophrenia and, furthermore, links inflammation to another prominent theory of the disorder [59].

## 4. How Might Inflammatory Cytokines Affect Brain Function?

One potential effect of MIA and the associated release of inflammatory cytokines is to activate microglia, the brain’s tissue-resident macrophages. These are cells that enter the brain early during neurodevelopment and that are long lived. As a result, changes in their activation patterns can have long-lasting consequences. While microglia can respond to infection or injury by initiating an inflammatory response and phagocytosing damaged neurons, so as to maintain CNS function, they also have an important role in the developing brain. Microglia have been shown to be involved in the development and maintenance of neural circuitry [60], with a role in guiding axons, pruning synapses, regulating myelination, and positioning interneurons [61,62]. Data from MIA models have shown that immune system activation during pregnancy has a number of different effects on microglial activation [63]. For example, in mice, MIA leads to a blunting in the microglial response in the adult offspring. This effect was associated with a decrease in the release probability of dopamine in the striatum, linking it to changes in a neurotransmitter system long associated with schizophrenia [64].

While disturbances in microglial activation could result in a general dysconnectivity in the developing brain, it is also clear that some impacts are specific to particular cell types. For example, Park et al. reported that, in culture, activated microglia affected developing cortical interneurons by impairing mitochondrial function, disrupting dendritic arborisation, synapse formation, and neurotransmitter release in these cells [65]. Inhibitory interneurons release the inhibitory transmitter gamma-aminobutyric acid (GABA) and are a key part of the microcircuitry of the cerebral cortex, with a primary function of limiting and shaping the activity of principal cells. Although there are a large number of different types of GABAergic interneurons in the brain [66,67,68], with a variety of associated characteristics [69], a considerable body of the schizophrenia research literature has tended to focus on the subset of inhibitory interneurons that are found throughout the brain and express the protein parvalbumin (PV) [70,71]. In the neocortex and hippocampus, most PV interneurons are capable of generating action potentials at a high rate due to their short actional potential duration and refractory period. These fast-spiking interneurons have projections onto pyramidal neurons, and because of their fast-firing characteristics, they are able to provide precise inhibitory control over principal cells via both feedforward and feedback connectivity.

The initial impetus for research into the role of GABAergic interneurons in schizophrenia originated in studies that showed a dysfunction in these neurons in postmortem analyses of patients with the disease [72]. These postmortem studies have consistently revealed reduced expression of GABA-related markers, such as the enzyme glutamic acid decarboxylase (GAD67) and parvalbumin (PV), in the prefrontal cortex of individuals with schizophrenia [73,74]. These deficits are particularly prominent in the fast-spiking PV-positive interneurons [75,76]. Consistent with a role for these GABAergic neurons in some of the cognitive functions that are disrupted in schizophrenia, magnetic resonance spectroscopy studies have reported correlations between symptom severity and cognitive deficits, and decreased GABA levels, in the brain of patients with schizophrenia [77,78,79].

## 5. How Inflammatory Cytokines Affect GABA

Previous research shows that inflammatory cytokines can affect GABAergic function in a number of ways. During critical periods of neurodevelopment, GABAergic interneurons undergo extensive maturation, including the establishment of synaptic connections; the acquisition of specific molecular markers, such as parvalbumin (PV); and the refinement of inhibitory circuits that regulate cortical excitability. Disruptions in these processes, mediated by inflammatory cytokines, can lead to enduring deficits in inhibitory neurotransmission and network dysfunction. Disrupting GABA signalling during early development can in turn alter cellular migration and cortical architecture [80]. The results of many animal-model studies show that maternal immune activation results in long-lasting deficits in GABAergic interneuron function, including reduced PV expression, paralleling findings in schizophrenia [81,82]. In particular, IL-6 has been implicated in reducing the expression of GAD67, the enzyme responsible for synthesising GABA, thereby diminishing inhibitory signalling during critical developmental windows [83]. Pro-inflammatory cytokines such as interleukin-1β (IL-1β), tumour necrosis factor-alpha (TNF-α), and interferon-gamma (IFN-γ) have also been shown to modulate GABAergic signalling by altering GABA receptor expression, inhibiting GABA synthesis, and disrupting inhibitory synaptic transmission [84]. These developmental disruptions can lead to persistent deficits in cortical inhibition and cognitive function, mirroring the neuropathological and behavioural abnormalities observed in schizophrenia. If changes in GABAergic function occur early on in development, they may underlie some of the symptoms observed in the prodrome, before the initial psychotic episode [85], or in some of the neurological soft signs that are observed from early life and that are predictive of later development of the disorder [86,87]. Furthermore, these early changes in GABA may be a ‘first hit’ that increases vulnerability to a second hit that occurs later in development and triggers schizophrenia.

The effects of maternal immune activation on GABAergic interneuron development are dependent on a number of factors, including the timing of exposure relative to the stage of gestation [88,89]. For example within the hippocampus, effects on PV interneurons depend on an interaction between sex, time of exposure during gestation, and subregion [90], while markers for somatostatin receptor 2, a receptor found on interneurons that release the neurotransmitter somatostatin, are timing dependent in the cortex [91]. These temporal effects highlight the vulnerability of GABAergic interneurons to inflammatory insults during distinct phases of neurodevelopment. Furthermore, the interaction between cytokines and other developmental factors, such as oxidative stress and mitochondrial dysfunction, can exacerbate the impact on interneuron maturation [65,92,93].

The interplay between inflammation and GABAergic dysfunction is also evident in adults, as individuals with schizophrenia often exhibit elevated levels of peripheral and central inflammatory markers, which correlate with cognitive deficits and reduced GABAergic activity [94,95]. In the adult brain, TNF-α, for example, has been shown to modulate synaptic scaling by increasing the surface expression of excitatory AMPA receptors while simultaneously decreasing inhibitory GABAergic signalling, leading to an imbalance in neuronal excitability [96]. This cytokine-mediated disruption of synaptic homeostasis may contribute to the hyperexcitability and network dysregulation observed in conditions such as schizophrenia. Moreover, chronic exposure to inflammatory cytokines can induce oxidative stress and mitochondrial dysfunction in GABAergic interneurons, further compromising their ability to maintain an inhibitory tone [97].

## 6. How Changes to GABA Could Alter Neural Oscillations

How is it that changes in GABAergic function could produce the cognitive and behavioural effects observed in schizophrenia? One proposal is that GABAergic dysfunction results in an imbalance between excitatory and inhibitory neurotransmitter systems, which leads to downstream effects. For example, in one prominent hypothesis, schizophrenia is linked to a downregulation of NMDA-subtype excitatory glutamate receptors that are located on GABAergic interneurons. This change results in a decrease in the activity of these interneurons, which leads to hypofunction in the inhibitory component of the circuit [98]. As a result of the excitatory/inhibitory balance in the circuit shifting towards excitation, there are problems controlling the gain of information flow within brain circuits [99]. While this hyperexcitability hypothesis might seem to explain some of the positive symptoms of schizophrenia, such as hallucinations and delusions, further detail is required to account for the negative and cognitive symptoms of the disease. It is also unclear how these changes would produce a differential effect to that which occurs in other disorders such as epilepsy, where changes in the excitation/inhibition balance are also implicated [100]. While there are several other potential mechanisms via which changes in GABAergic function could produce the signs and symptoms of schizophrenia, for example, by modulating dopamine activity [101], for the purposes of this review, we will focus on a model where GABAergic dysfunction leads to compromised information storage and processing that is more subtle than can be explained by a simple hyperexcitability hypothesis. This proposal, which has been explored in some depth previously, is the hypothesis that changes in GABAergic function directly affect the brain’s ability to synchronise information across space and time. This would compromise many aspects of brain function, for example, the ability to integrate information from different sensory areas, the ability to detect cause-and-effect relationships, and difficulty in coherent motor planning, effects that are all evident in schizophrenia. A synchronisation problem has previously been linked to changes in the oscillatory mechanisms that underlie brain rhythms [102,103,104]. Rhythmic activity in the brain is organised into distinct frequency bands, particularly, the theta (~2–10 Hz), beta (~12–30 Hz), and gamma (~30–90 Hz) bands [105,106,107]. One function of this oscillatory activity is to synchronise neural activity, both within local regions and across wider brain networks [108]. For example, it is known that several cognitive and behavioural tasks associated with prefrontal and hippocampal regions require the precise spike timing of pyramidal neurons in relation to rhythmic oscillations of the local field potential [105,109,110,111]. Neural oscillations also have a role in parcellating neural processing into temporally constrained blocks [112,113] that may allow for the separation of distinct stages of processing [114].

A key factor that underlies the generation and modulation of many of these oscillations is the relationship between excitatory and inhibitory neural activity. Both experimental and computational research suggests that incoming neuronal activity interacts with local circuitry to generate fast oscillations, such as gamma- and the high-frequency ripples that are associated with memory consolidation in the hippocampus [115,116,117]. These excitatory/inhibitory interactions can be observed in a number of different brain areas. For example, PV interneurons have a critical role in synchronising gamma oscillations in the primary visual cortex [118], and gamma oscillations nested within theta-frequency activity in the medial entorhinal cortex are dependent on feedback inhibition [119], as is coherent activity in the hippocampus [115]. While gamma-frequency activity is associated with active information processing, theta-frequency activity in the hippocampus is associated with memory and learning [120]. Theta is also shaped by a series of excitatory and inhibitory events that occur at particular phases of the theta cycle [121,122,123], and this activity is under the control of GABAergic pacemaker neurons located in the medial septum [124]. Deletion of the ErbB4 receptor in fast-spiking PV interneurons leads to impairments in hippocampal–prefrontal synchrony at theta frequencies in a mouse model [125]. Critically, this receptor is a part of a signalling pathway that is critical for the development of neocortical and hippocampal inhibitory circuits [126]. Furthermore, it has also been linked to neuroinflammation and subsequent changes in theta and gamma oscillations [127].

## 7. Links Between Neural Oscillations and Schizophrenia

Given the important role of oscillatory activity in synchronising information across space and time in the brain, it is possible that changes in GABAergic function, as occurs in schizophrenia, could compromise this capability [128]. Consistent with this proposal, a number of studies have reported that schizophrenia is associated with a disruption of neuronal oscillations in several frequency bands [102,103,129,130]. Initial research showed that activity in the 40 Hz-frequency band, elicited by auditory stimulation (auditory steady-state responses; ASSRs), had reduced power and phase synchronisation in individuals with schizophrenia [131,132]. ASSR impairments were also evident in patients with first-episode psychosis, as well as participants at high risk for psychosis, indicating that these effects were unlikely to be a result of medication or psychosis itself, and may have existed for some time prior [133]. It was subsequently shown that individuals with schizophrenia exhibited reduced 40 Hz-range, EEG gamma band responses; delayed phase coherence; and reductions in interhemispheric coherence [134]. Again, several of these changes were shown to occur in individuals at risk for schizophrenia or psychosis [135,136]. Investigations of oscillations during speech and language processing also revealed changes in delta- and theta-frequency band synchrony between fronto-temporal regions during talking vs. listening in people with schizophrenia [137]. Hirvonen et al. also reported that patients with schizophrenia were characterised by a reduction in gamma band oscillation amplitudes and a pronounced deficit in large-scale synchronization across brain networks, such as between the visual and frontal cortex [138]. Patients with schizophrenia also display a reduction in theta power and diminished theta-phase coupling between the mPFC and the medial temporal lobe compared to healthy control participants, and this effect was correlated with both memory performance and abnormal GABAA receptor expression in the schizophrenia group. Similar findings have been observed in animal models of the disorder, suggesting that the theta rhythm in particular may be important for long-range functional connectivity between the prefrontal cortex and the hippocampus and, by extension, cognitive tasks that require memory or executive function [139,140]. Inflammation has been linked to these effects, consistent with a role in the development of schizophrenia via an impact on oscillations. For example, elevations of TNF-alpha were linked to blunted alpha- and beta-frequency oscillations, as measured by MEG, when participants were performing an abstract reasoning task [141]. In an animal model, Mamad et al. demonstrated that an inflammation-inducing interperitoneal injection of LPS resulted in a reduction of theta-frequency activity and an increase in lower-frequency delta activity in the hippocampus [142]. Similarly, Hirao et al. have shown that network oscillations are altered in brain slices obtained from the anterior cingulate cortex in LPS-treated mice, a preparation that isolates inflammatory effects on oscillatory mechanisms from changes in behaviour that themselves may result in altered oscillatory activity [143].

## 8. How Disruptions in Oscillatory Activity Could Lead to Disorganisation Symptoms

Disturbances in the synchronisation of neural activity, as might occur when oscillations are disrupted, may result in the disorganisation of experience and memory. In schizophrenia, this may be apparent in disorganised speech and thought, symptoms that have been proposed to reflect deficits in integration [144], disruptions in excitatory/inhibitory balance, and oscillatory dysconnectivity [145]. Changes in synchronisation may also underlie the temporal and sequence processing deficits that are well documented in schizophrenia. This includes disturbance in the judgement of temporal order and duration [146,147], predictive timing [148], transitive inference [149,150], and sequence learning [151,152,153]. Similar deficits have also been observed in first-degree relatives and other at-risk individuals during the prodromal phase [154], and they are independent of other cognitive impairments [146]. The ubiquity and early expression of these types of timing and sequencing deficits suggest that they may be a primary feature of the disorder, occurring prior to the first psychotic episode, and a potential trait marker for schizophrenia [155].

Although timing and sequencing processes are ubiquitous through the brain, many of the particular processes that are disturbed in schizophrenia have been linked to the hippocampus, particularly as they relate to episodic memory [156,157,158]. Previous evidence from both in vivo and post-mortem studies indicates that abnormalities in the hippocampus are a feature of schizophrenia and autism, in both cases disorders where risk has been linked to exposure to inflammation during neurodevelopment [159,160,161,162,163]. Studies of the hippocampus as it relates to both schizophrenia and autism have reported both structural and functional alterations in this region [159,160]. Consistent with this region having a role in timing and sequencing processes, disruptions in hippocampal activity have been suggested to have a role in the generation of disorganisation symptoms in schizophrenia [164,165].

As a model of the effect of inflammatory processes, maternal immune activation has been shown to impact both oscillatory processes in the hippocampus and their connections to other regions [166,167,168]. For example, studies using both the poly (I:C) and the methylazoxymethanol acetate (MAM) model have independently demonstrated changes in GAD67 expression and PV+ interneuron activity in hippocampal regions [169,170], and in both cases, these reductions were accompanied by aberrant neural synchrony between the hippocampus and medial prefrontal cortex. Furthermore, dysregulated oscillatory networks also appear to compromise the integrity of hippocampal projections to other subcortical areas, such as the LS, the striatum, and the ventral tegmental area [157,171,172,173]. These latter regions are important for dopaminergic modulation, and so disruptions to the coordinated spiking activity of upstream neurons are likely to exert effects on subcortical dopaminergic activity. In turn, striatal dopaminergic concentrations have been shown to strongly influence the synchronisation of GABAergic micro-circuits in a computational model, suggesting that the relationship might be reciprocal, and that dopamine might have a wider modulatory role in the maintenance of functional connectivity [174].

Previous work has associated changes in brain oscillations to disorganisation symptoms in schizophrenia [175]; however, it will be important to demonstrate mechanistically how this might lead to changes in the neural processing of information. One form of neural synchrony observed in the hippocampus (and elsewhere) that has been consistently linked to a neural mechanism underlying sequence learning for more than three decades [176,177] depends on the phase relationship between neuron firing and the ‘background’ local field potential and is known as phase precession. Here, the hippocampal theta rhythm serves as a reference signal, against which the phase of firing of hippocampal principal cells is related to the location of the animal in space.

Theta-phase precession was initially observed in CA1 “place” cells. When the firing of these cells was referenced to the underlying theta-frequency LFP oscillation, it was noted that the phase of firing changed systematically from later to earlier phases as an animal moved through that cell’s place field [176,177]. As a result, the firing phase of a cell, relative to the theta cycle, was shown to provide information about where the animal is located within a place field, over and above that of the firing rate code [157,178,179]. Critically, when several cells with overlapping place fields generate phase precession, the combined activity produces an emergent phenomenon known as a ‘theta sequence’ [180]. During a theta sequence, recently experienced episodes that have occurred at behavioural timescales are preserved and compressed into a single theta cycle (~120 ms). During this time-compressed period, synaptic plasticity mechanisms can operate to link the neurons representing that episode [176,181]. As a result, theta sequences have received considerable interest as a potential mechanism underlying sequential memory encoding and storage [113,176,182,183,184], a proposal that aligns with findings that the developmental emergence of theta sequences coincides with the maturation of hippocampal memory in rodents [185]. Phase precession and theta sequences have also been observed to occur in tasks that require goal planning and decision making [186,187,188], and in paradigms that do not include a spatial component, suggesting an involvement in processes outside the spatial domain [189,190,191,192]. Furthermore, several studies have reported phase precession in single-cell recordings in human participants [193]. In one recent example, this activity was linked to memory processes in human participants watching and remembering movie clips [194], with indications that the process may be associated with the encoding of event boundaries [195,196].

**Figure 1:**
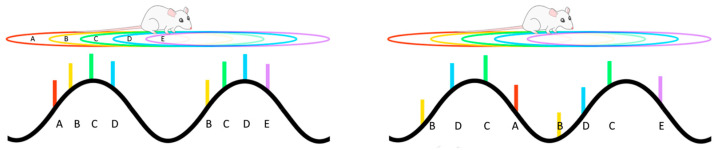
As an animal moves along a trajectory (**top left**), multiple hippocampal place cells will be simultaneously active and responding to particular overlapping regions of space (their place fields A–E). At any particular moment, the currently active cells will fire (coloured bars) at a particular phase of the underlying theta rhythm (**bottom left**), with their firing phase being sequentially related to the order of the place fields. Maternal immune activation results in a disordering of this phase coding (**bottom right**), so that the phase sequence no longer matches the spatial sequence of place fields.

If theta sequences provide the biophysical scaffolding that supports the encoding and storage of temporally extended memories, perhaps via the construction of mental maps, an important component of both episodic memory and decision making [197], then a disruption of this system could result in disorganisation of thought, as observed in schizophrenia [106]. Since the phase of firing of hippocampal cells during phase precession is tightly regulated by phasic inputs [198], with PV+ interneurons playing a crucial role [125,169,170,184,199,200], can manipulations that trigger inflammation sufficient to have an effect on the development of these interneurons and their connections alter phase precession? One recent study has investigated whether phase precession is altered in the MIA model, and here it was shown that, compared to control animals, neurons in the hippocampal CA1 region of MIA animals displayed considerably more variation in the starting phase of the precession as the animal entered the cell’s place field [166]. Since phase precession otherwise continues normally, but with a random phase offset on each precession phase trajectory, there are major consequences for theta sequences [166], as they are reproduced in a disordered manner that does not match the sequential ordering of experience (Figure 1). While a change in phase precession in individuals with schizophrenia has not yet been described, it is of interest to note that there are alterations in the neural replay that appear to underlie the representation of sequential relationships [153].

Hippocampal phase coding has been associated with the sequential integration of sound and odour cues [201], as well as internally generated states [202,203]; therefore, variation in the phase offset of precession in schizophrenia could potentially underlie the disintegration of experience and memory that is apparent in the disease [166]. An additional effect of increased starting phase variability is that the sequential spiking that occurs within each consecutive theta cycle is less clustered, and as a result, event sequences may overlap and merge [166]. This could contribute to disintegration of event boundaries [106,204], a phenomenon observed for both lower- and higher-order levels of processing among individuals with schizophrenia [205,206].

While we have described here how inflammation can lead to disorders of rhythmic activity, with a particular focus on the hippocampus, it is also worth noting that this region not only codes for space [207] but also for time. Investigations of ‘time’ cells, initially discovered in the hippocampus proper [208] and more recently in the medial entorhinal cortex [209], have shown that these cells represent the flow of time by firing at specific epochs during an episode, such that the population of neurons codes the entire temporal space of tasks that may last for many seconds [210]. Since timing and sequencing processes are disturbed in schizophrenia, and since these processes have previously been linked to the hippocampus [158,182,210], it will be of interest to explore whether hippocampal time cells are affected in the disorder. Changes in interneuron function may affect their activity, as it has been proposed that the mechanisms are dependent on recurrent neural connections that feed information back into a circuit with some delay [211]. The inclusion of delays in such circuits will likely depend on oscillations that provide some temporal distance between bursts of neural activity. Consistent with this model, time cell firing appears to be tied to rhythmic theta activity cycles [192]. These findings suggest that inflammation-mediated changes in GABAergic inhibition might be disruptive to time cell activity, and while time cells are difficult to examine in humans (although see [212]), they can be examined in detail in animal studies. At the present moment, however, there have been no investigations of these cells in an animal model of schizophrenia. This is clearly an avenue for future research.

## 9. Conclusions

**Figure 2:**
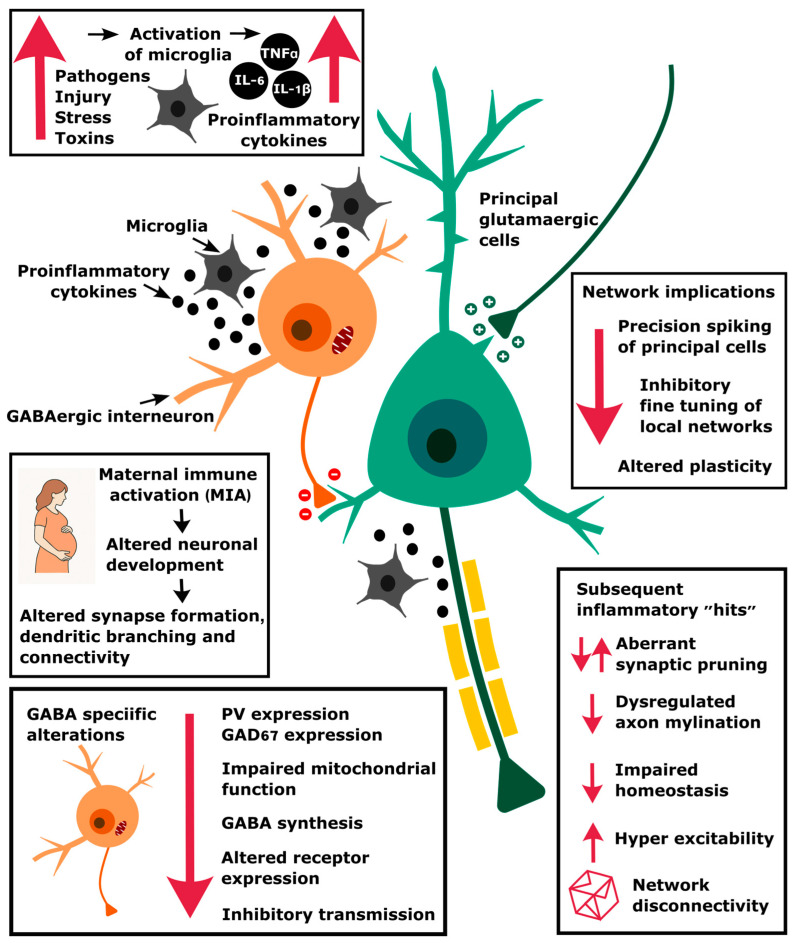
An illustration of how inflammation, triggered by maternal immune activation and other stressors, can result in changes at the molecular level that compromise GABAergic function and network circuitry. This then affects the precision of spike timing, particularly during oscillatory activity, which alters plasticity and synchrony mechanisms.

Our ability to develop a sophisticated understanding of the biological basis of schizophrenia has been limited, in part, because of the complexity of the disorder. This has resulted in difficulties in linking changes in basic mechanisms, such as neurotransmitter systems, to high-level psychological symptoms [213]. These and related difficulties have meant that drug treatment strategies for schizophrenia have progressed only modestly over the last 40 years [213,214]. As a result, currently available treatments, while being somewhat effective against psychosis, are only efficacious in about half of patients, and often have little impact on the cognitive and negative symptoms [215]. This review has attempted to link initiating processes with mechanistic outcomes by focussing on the role of inflammation in disrupting inhibitory interneuron function in the brain and how this has consequences for the oscillatory activity that underlies timing, sequencing, and synchronisation in the brain (Figure 2). Disturbance in these latter processes has previously been proposed as a primary feature of schizophrenia and a potential trait marker for the disorder [155]. If changes in GABAergic interneuron function are an important mediator of the effects of inflammation on brain oscillations, then this transmitter system is an obvious target for therapeutic intervention. This can be attempted by manipulation of GABA systems directly or via modulation of the glutamatergic systems that activate interneurons [216,217]. Previous research in this area has, however, resulted in mixed results [218], with a need for better targeting of the errant systems, a project in which there is continued interest [219,220,221,222,223]. In this regard, the hippocampus will be a useful model system in this approach, as oscillating rhythms underlie much of the processing that occurs in this region. Furthermore, it has previously been implicated in schizophrenia [160,224,225,226], it is a region where disturbance could underlie some of the cognitive deficits observed in the disorder [227,228], and it is a location where defective GABAergic neurotransmission could be a critical factor in this dysfunction [162,219,229,230,231].

## Data Availability

Not applicable.

## Abbreviations

The following abbreviations are used in this manuscript:

MIA	Maternal Immune Activation
GABA	gamma-aminobutyric acid
GAD67	glutamate decarboxylase
NMDA	N-methyl-D-aspartate
PV	Pavalbumin
